# Genetic Diversity, Reassortment Patterns, and Antigenic Characterization of Two H9N2 Avian Influenza Viruses Isolated from Quails in China

**DOI:** 10.3390/v18080919

**Published:** 2026-08-21

**Authors:** Yutao Teng, Peidong Li, Fuyou Zhang, Wanting Zhou, Xue Wang, Hao Zhu, Qingqing Song, Zhaoyang Li, Chunguo Liu

**Affiliations:** Group Biological Products R&D Center, Shandong Sinder Technology Co., Ltd., Qingdao 266101, China; tengyutao@sinder.cn (Y.T.); lipeidong@sinder.cn (P.L.); zhangfuyou@sinder.cn (F.Z.); zhouwanting@sinder.cn (W.Z.); wangxue@sinder.cn (X.W.); zhuhao@sinder.cn (H.Z.); songqingqing@sinder.cn (Q.S.); lizhaoyang@sinder.cn (Z.L.)

**Keywords:** avian influenza virus, H9N2 subtype, quail, gene reassortment, antigenicity

## Abstract

H9N2 avian influenza viruses pose a persistent zoonotic risk owing to their broad host adaptability. Between 2024 and 2025, two H9N2 isolates (ZHY1022B2 and ZHY0417A11) were recovered from quail flocks in Hebei, China. Phylogenetic analysis clustered their HA genes within the dominant B4.7.2 subclade, but diverged into two subgroups (B4.7.2.2 and B4.7.2.1). Of particular interest was ZHY0417A11, which displayed a multigenic reassortment pattern—its PB1 originated from an H3N8 virus, PA and PB2 from H3N3, NS from H10N3, and the M gene was nearly identical (99.32%) to a human H3N8 isolate, whereas the remaining HA, NA and NP segments maintained the H9N2 backbone. Despite the presence of the HA mammalian-adaptive markers (H191N, A198V and Q234L), both strains showed marked antigenic drift from the vaccine strain SS (BJ/94-like; R < 0.5), while retaining reactivity with currently circulating field strains. These findings argue for heightened vigilance in quail populations, given their role as mixing vessels, and highlight the limitations of current vaccine matching in light of ongoing H9N2 evolution.

## 1. Introduction

H9N2 avian influenza viruses (AIVs) have become globally endemic in poultry since their first detection in 1966 [1]. Although generally causing subclinical or mild respiratory infections, they predispose birds to secondary bacterial diseases and impose substantial economic burdens. More critically, H9N2 viruses have demonstrated direct zoonotic transmission to humans and repeatedly supplied internal gene segments to highly pathogenic strains such as H5N1 and H7N9 [2,3]. Their ongoing evolution, frequent reassortment, and expanding host range position them as pathogens of significant pandemic concern at the animal–human interface [4,5].

Quails are considered critical intermediate hosts for AIVs. Their respiratory and digestive epithelia co-express avian-type (SAα2,3-Gal) and human-type (SAα2,6-Gal) sialic acid receptors, a feature that facilitates mammalian adaptation [6]. Since the first quail AIV isolate in the 1960s [7], multiple subtypes—including H5N1, H9N2, and H10N8—have been detected in quail populations worldwide [4,8,9,10]. Accumulating evidence supports the view that quails act as efficient “mixing vessels,” promoting inter-subtype reassortment and accelerating host adaptation, thereby bridging wild-bird ecology and mammalian spillover [10,11,12].

China is the world’s largest producer and consumer of quail meat and eggs, with a commercial population exceeding 500 million in 2024. Production is geographically concentrated, with flock sizes surpassing 50 million in Henan, Anhui, Jiangsu, and Zhejiang Provinces alone [13]. Intensive rearing, high stocking densities, mixed poultry species farming, and frequent live-bird market trading create conditions conducive to inter-subtype reassortment, while the absence of commercially available vaccines against major pathogens further compounds the risk of epidemic outbreaks in the quail industry.

Epidemiological surveillance and molecular characterization of H9N2 AIVs have historically focused on chickens, ducks, and environmental samples from live-bird markets, leaving quails as a distinct host largely understudied. Despite sporadic reports of H9N2 infection in quail populations [14], comprehensive data on their genetic diversity, antigenic evolution, and host-adaptive mechanisms under current field conditions are still lacking [1,15,16]. Under sustained immune pressure, these viruses undergo continuous antigenic drift, leading to progressive mismatch with traditional vaccine strains (e.g., the BJ/94-like strain SS [17]), which may undermine vaccine efficacy and facilitate cross-species transmission.

To fill this knowledge gap and assess the cross-species transmission risk, we systematically characterized two H9N2 viruses isolated from quail flocks in Hebei Province (2024–2025). Luancheng District, Shijiazhuang City, is a major quail breeding base in North China, characterized by intensive farming and frequent trading [13], serving as a sentinel site for viral surveillance. Using whole-genome sequencing, phylogenetic analysis, molecular marker identification, and serological cross-reactivity assays, we aimed to (i) delineate the genetic diversity and reassortment patterns of these isolates, (ii) evaluate their antigenic relationship with vaccine and circulating strains, and (iii) assess their zoonotic potential. Our findings will inform early warning systems, vaccine strain selection, and targeted control strategies.

## 2. Materials and Methods

### 2.1. Outbreak and Sample Collection

An outbreak occurred on a commercial quail farm in Hebei Province in October 2024, with clinical signs including yellowish diarrhoea, intestinal haemorrhage, increased proventricular secretions, and decreased egg production. The affected flock comprised approximately 30,000 laying quails (235 days old), with morbidity and mortality of 10% and 0.5%, respectively. The birds were housed at high density but without contact with any other poultry species, and had been vaccinated at 18 days of age with an inactivated bivalent H5/H7 vaccine. Tissue samples (liver, spleen and lung) were collected from moribund birds and transported to the laboratory under cold-chain conditions.

A second outbreak was recorded on another quail farm in the same province in April 2025, primarily manifesting as dyspnoea and ulcerative lesions. The affected birds were 12 days old (approximately 60,000 in total), with morbidity and mortality of 5% and 0.3%, respectively. This farm also maintained single-species rearing without co-habitation with other poultry, and vaccination against H5/H7 had been administered at 7 days of age. Based on clinical presentation, tissue samples (liver, spleen, lung, kidney, trachea and caecal tonsils) were collected from diseased individuals, aseptically processed, and shipped to the laboratory under cold-chain conditions.

### 2.2. Sample Processing and Pathogen Identification

Tissue samples were homogenized and clarified by centrifugation, and total nucleic acids were extracted using a commercial kit (Vazyme, Nanjing, China). AIV subtypes were identified by a quadruple real-time RT-qPCR assay targeting the universal matrix gene and the H5, H7, and H9 subtype-specific sequences (Xinda Gene, Zhucheng, China; Cat. No. XD20250808). The thermal cycling protocol consisted of reverse transcription at 50 °C for 5 min, initial denaturation at 95 °C for 30 s, followed by 40 cycles of 95 °C for 10 s and 56 °C for 30 s. Samples with cycle threshold (Ct) values < 36 were considered positive and subjected to virus isolation. To rule out co-infections, all positive samples were further screened for Newcastle disease virus, infectious bronchitis virus, chicken anemia virus, avian reticuloendotheliosis virus, and *Salmonella* spp. using additional qPCR or RT-qPCR kits from the same supplier (Xinda Gene, Zhucheng, China).

### 2.3. Virus Isolation and Identification

RT-qPCR-positive tissue supernatants (0.2 mL) were treated with penicillin (3000 U/mL) and streptomycin (3000 μg/mL) for 1 h at room temperature, then inoculated into the allantoic cavity of four 9- to 11-day-old specific pathogen-free (SPF) embryonated chicken eggs (Beijing Boehringer Ingelheim Vital Biotechnology Co., Ltd. Beijing, China) per sample. Eggs were incubated at 37 °C and candled daily, with embryo deaths recorded. After 72 h, allantoic fluid was harvested from surviving embryos and from those that died ≥24 h post-inoculation. Hemagglutination activity was determined using 1% chicken red blood cells, and the viral subtype was confirmed by hemagglutination inhibition (HI) assays with subtype-specific antisera against H3, H5, H6, H7, and H9 AIVs. Hemagglutinin-positive fluids were blind-passaged three consecutive times, and the final virus stocks were aliquoted and stored at −80 °C until use.

### 2.4. Whole-Genome Sequencing and Molecular Genetic Evolution Analysis

Viral RNA was extracted from infected allantoic fluid using a commercial kit (Vazyme, Nanjing, China). The eight gene segments were amplified by one-step RT-PCR (Accurate Biotechnology, Changsha, China) with universal primers [18]. Purified PCR amplicons were Sanger-sequenced (Tingke, Qingdao, China). Sequences were assembled using SnapGene v6.0 (GSL Biotech) to obtain complete coding sequences. Nucleotide and amino acid identities were calculated with BioEdit v7.2.5 using the Clustal W algorithm [19]. Sequences have been deposited in GenBank (accession numbers are listed in the Data Availability Statement). Phylogenetic trees were constructed using the maximum likelihood method with the GTR + G + I model [20] under the H9 classification scheme proposed by Fusaro et al. [21]; branch support was assessed with 1000 bootstrap replicates. Tree visualization and annotation were done with tvBOT (Tree Visualization By One Table, https://www.chiplot.online/tvbot.html, (accessed on 10 May 2026)) [22]. For reassortment analysis, the most likely subtype origin of each gene segment was inferred by BLASTn searches against the NCBI nucleotide database (https://blast.ncbi.nlm.nih.gov/Blast.cgi, (accessed on 18 May 2026)), and the results were summarized graphically. N-glycosylation sites were predicted with NetNGlyc 1.0 Server (https://services.healthtech.dtu.dk/services/NetNGlyc-1.0/ (accessed on 20 May 2026)), and HA residue numbering follows the full-length, immature H9 HA precursor.

### 2.5. Cross-HI Assays

Four-week-old SPF chickens were immunized subcutaneously with 0.3 mL of inactivated oil-emulsion vaccine (each dose contained 0.1 mL of antigenic solution with a hemagglutination titer of 8 log_2_) to raise antisera. Sera were collected two weeks later and stored at −20 °C. Reference antisera against the vaccine strain A/Chicken/Guangdong/SS/94 (H9N2) (SS, BJ/94-like) and circulating field strains A/Chicken/Liaoning/HLD/ZGS0205/2021(H9N2) (ZGS, B4.7.2.2) and A/Chicken/Guangxi/YL/LTD0723/2021(H9N2) (LTD, B4.7.2.1) were provided by Shandong Sinder Technology Co., Ltd. (Qingdao, China). The homologous HI titers (log_2_) for ZHY1022B2, ZHY0417A11, SS, ZGS, and LTD were 9, 11, 11, 12, and 10, respectively. Cross-HI assays followed the WOAH Manual of Diagnostic Tests and Vaccines for Terrestrial Animals (Chapter 3.3.4). The antigenic correlation coefficient (R) was calculated using the Archetti–Horsfall formula [23,24,25]; R ≥ 0.5 was considered indicative of minor antigenic difference, whereas R < 0.5 indicated substantial divergence, and values exceeding 1.0 were normalized to 1.0.

## 3. Results

### 3.1. Virus Isolation and Identification

Two H9 viruses, ZHY1022B2 (October 2024) and ZHY0417A11 (April 2025), were recovered from quail tissues sampled on two farms in Hebei Province. Both isolates were confirmed as H9 subtype by RT-qPCR and HI assays with subtype-specific antisera, and allantoic fluids from infected eggs consistently reached hemagglutination titers of ≥8 log_2_.

### 3.2. Phylogenetic and Genetic Analysis

**Figure 1:**
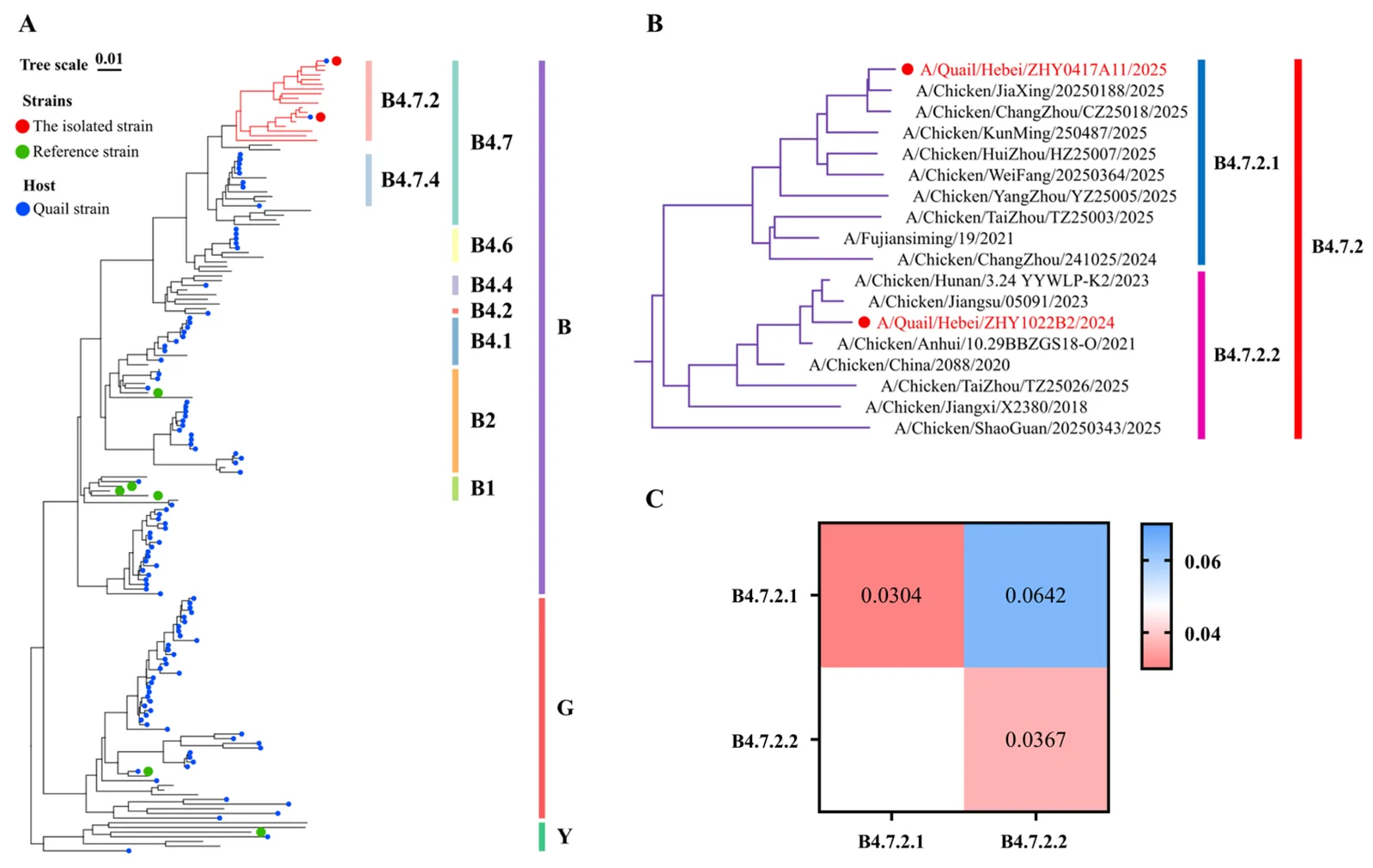
Phylogenetic tree based on HA gene of H9N2 avian influenza viruses. (**A**) Overall tree showing lineages B, G, and Y. (**B**) Expanded view of subclade B4.7.2 showing subclusters B4.7.2.1 and B4.7.2.2. (**C**) *p*-distance matrix between the branches of B4.7.2.1 and B4.7.2.2. The two isolates from this study are indicated by red dots.

Phylogenetic analysis of the HA gene revealed that both isolates fell within lineage B, subclade B4.7.2 (Figure 1A), which currently predominates in Chinese poultry. However, they diverged into distinct subgroups, with ZHY1022B2 assigned to B4.7.2.2 and ZHY0417A11 to B4.7.2.1 (Figure 1B). Their HA sequences shared 93.4% nucleotide and 95.0% amino acid identity, and the mean pairwise distance between the two subgroups was 0.0642 (Figure 1C). For ZHY1022B2, the closest relative was A/chicken/Hunan/3.24 YYWLP-K2/2023 (H9N2), sharing 98.93% nucleotide and 99.82% amino acid identity. In ZHY0417A11, the highest nucleotide identity (97.68%) was with A/environment/Xiamen/01/2021 (H9N2) (Table 1), while the closest amino acid match (also 97.68%) was A/chicken/China/YL/2022 (H9N2) (Table S1).

**Figure 2:**
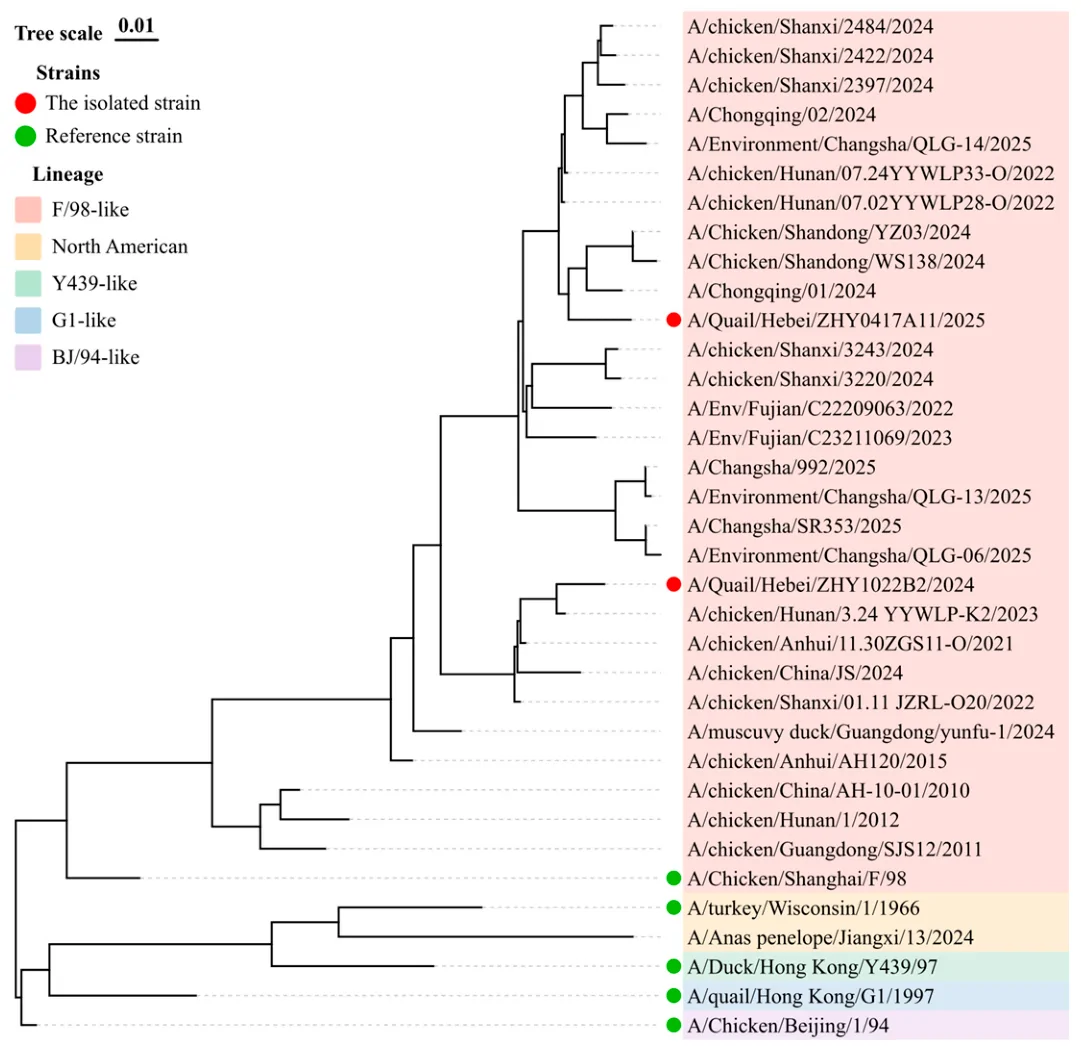
Phylogenetic tree based on NA gene of H9N2 avian influenza viruses. The two isolates from this study are indicated by red dots.

For the NA gene, both viruses were assigned to the F/98-like lineage (Figure 2) and were most closely related to recent chicken isolates from Hunan. The two isolates exhibited 97.3% nucleotide and 98.5% amino acid identity with each other (Table 1 and Table S1).

**Figure 3:**
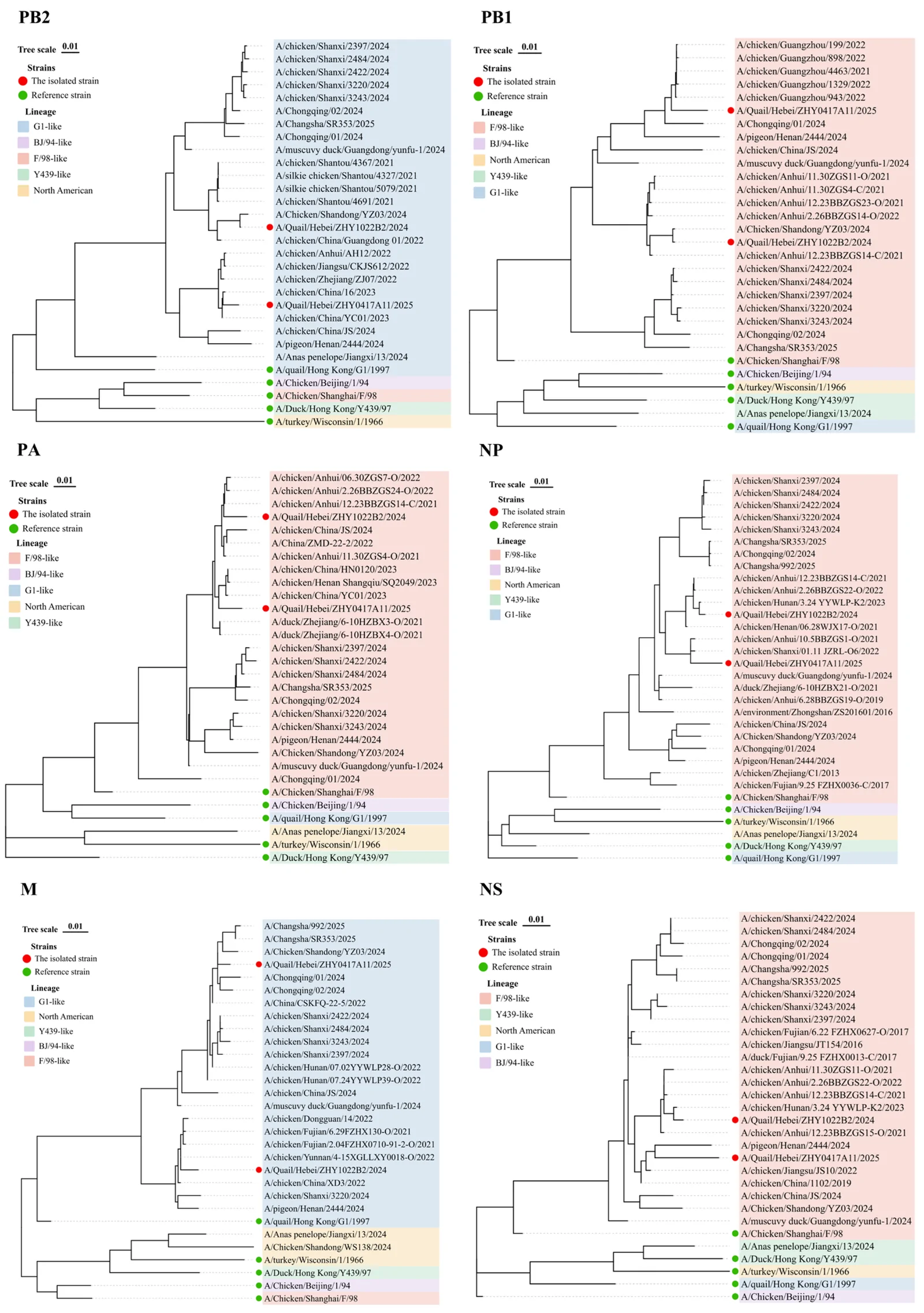
Phylogenetic tree based on the internal genes of H9N2 avian influenza viruses. The two isolates from this study are indicated by red dots.

Phylogenetic analysis of the six internal gene segments (PB2, PB1, PA, NP, M, and NS) revealed distinct lineage distributions (Figure 3). ZHY1022B2 carried PB2 and M in the G1-like lineage, while PB1, PA, NP, and NS grouped within the F/98-like lineage. In contrast, the internal genes of ZHY0417A11 showed a more complex mosaic, with segments tracing to H3N3, H3N8, H9N2, and H10N3 origins (see Section 3.3 and Table 1 for details).

### 3.3. Reassortment Analysis

**Figure 4:**
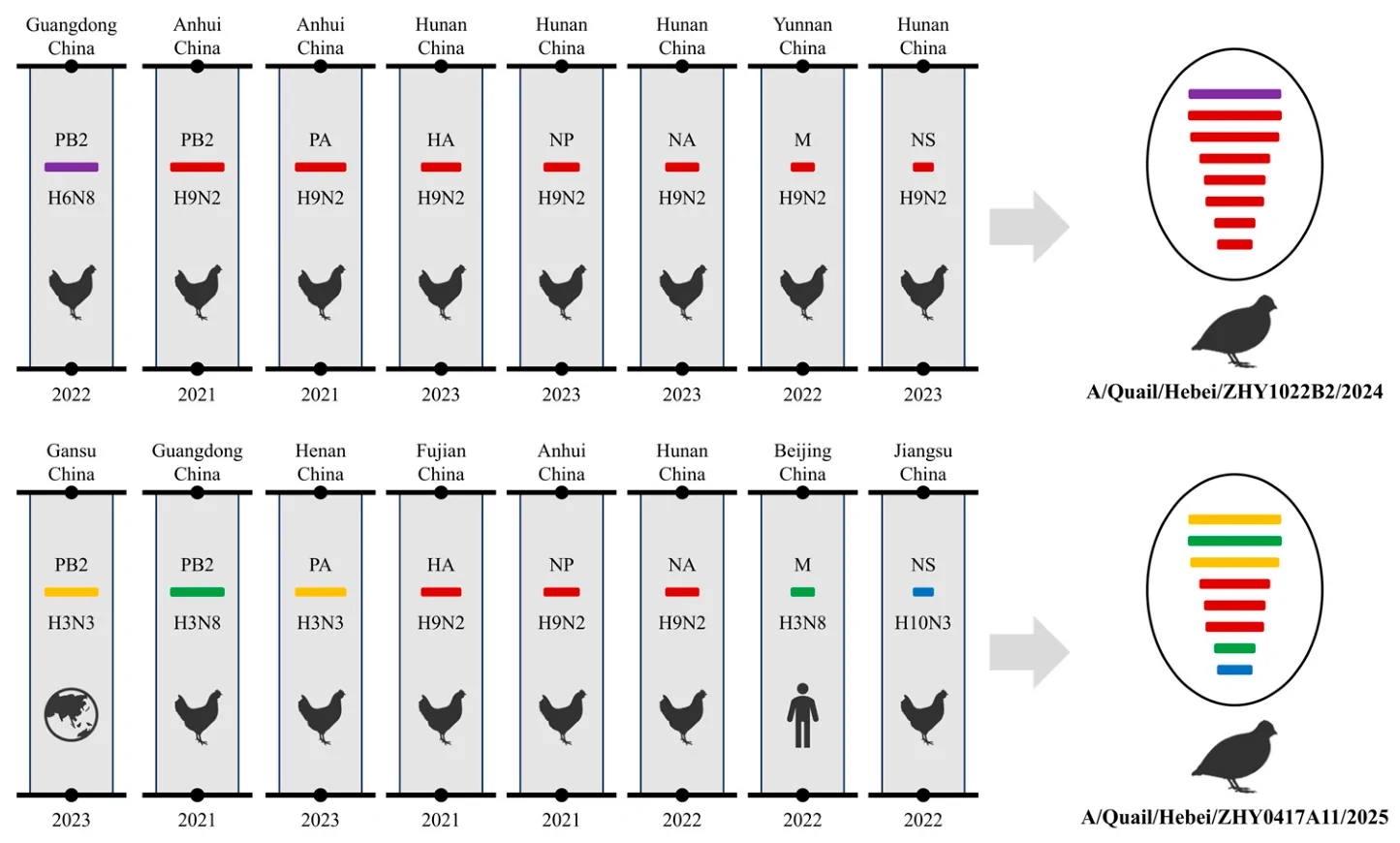
Diagram of reassortment analysis showing the gene segment origins of ZHY1022B2 and ZHY0417A11. Different colors represent different subtype origins. The M gene of ZHY0417A11 is highlighted to indicate its human-origin H3N8 source.

Based on sequence identity (Table 1) and phylogenetic clustering (Figure 3), the genomic origins of the eight segments were summarized in a schematic diagram (Figure 4). ZHY1022B2 carried a largely H9N2-backbone genome, with the exception of its PB2 gene, which was derived from an H6N8 virus (A/chicken/China/Guangdong_01/2022, 98.46% nucleotide identity); the remaining five internal genes (PB1, PA, NP, M, and NS), together with NA, were all of H9N2 origin. In contrast, ZHY0417A11 displayed a more complex reassortant architecture, involving at least four different subtype donors. Its PB2 and PA genes originated from H3N3 viruses (98.37–98.73% identity with A/chicken/China/YC01/2023 and A/chicken/Henan Shangqiu/SQ2049/2023, respectively); PB1 was most closely related to H3N8 (97.93% identity with A/chicken/Guangzhou/4463/2021); NP traced to an H9N2 virus (98.03% identity with A/chicken/Anhui/11.30ZGS1-C/2021); the M gene exhibited the highest identity (99.32%) to a human-origin H3N8 strain (A/China/CSKFQ-22-5/2022), although it phylogenetically clustered within the G1-like lineage; NS was derived from H10N3 (97.37% identity with A/chicken/Jiangsu/JS10/2022).

### 3.4. Molecular Characterization of Key Amino Acid Sites

#### 3.4.1. HA Protein

Compared with the classical vaccine strains SS and F/98, both isolates carried substitutions at multiple residues within the HA receptor-binding domain (RBD; H9 numbering) (Table 2). In the left edge region (positions 146–150), both shared K149N, and ZHY1022B2 additionally carried A150T, whereas ZHY0417A11 retained 150A. Within the internal RBD, both isolates shared T163N and A198V, while positions 191, 202, and 203 remained unchanged (N, L, and Y, respectively). In the right edge region (positions 232–237), both harboured Q234L and Q235M; ZHY0417A11 also had N232H, while ZHY1022B2 retained 232N, matching the reference strains. The potential N-glycosylation motif (N-X-S/T) at position 163 was conserved in both isolates.

At the HA antigenic sites (Table 3), relative to SS, both isolates shared S145D, D153G, A168N, T200R, and N201T. Additionally, ZHY1022B2 carried G90E and N167G, whereas ZHY0417A11 displayed G90D and retained 167N.

#### 3.4.2. NA Protein

The NA proteins of both isolates consisted of 466 amino acids, with a three-residue deletion (Δ63–65) in the stalk region. Potential N-glycosylation sites were predicted at positions 69 (NST), 86 (NWS), 146 (NGT), 200 (NAT), 234 (NGT), and 368 (NGS/NSS) in both isolates; ZHY0417A11 had an additional site at 402 (NWS), which was absent in ZHY1022B2. Within the hemadsorbing site (positions 366–373), both carried D369G/S. At positions 399–404, ZHY1022B2 exhibited D401V and N402D, and both isolates shared T434P.

#### 3.4.3. Internal Proteins

Amino acid residues in PB2, PB1, PA, NP, NS1, and M previously linked to mammalian adaptation, polymerase activity, or virulence were compiled in Table 4 [26,27,28,29,30,31,32,33,34,35,36,37,38,39,40,41,42,43,44,45,46]. Both isolates shared identical substitutions at multiple sites: NS1-42S, -138F, and -149A; PB2-89V, -292V, and -588V (with E retained at 627); PA-224P, -356R, -383D, and -672L; and PB1-3V and -13P. PB1-I368V was present only in ZHY1022B2, while ZHY0417A11 retained I at that position. Both isolates carried NP-105V and -184K.

### 3.5. Antigenic Analysis

**Figure 5:**
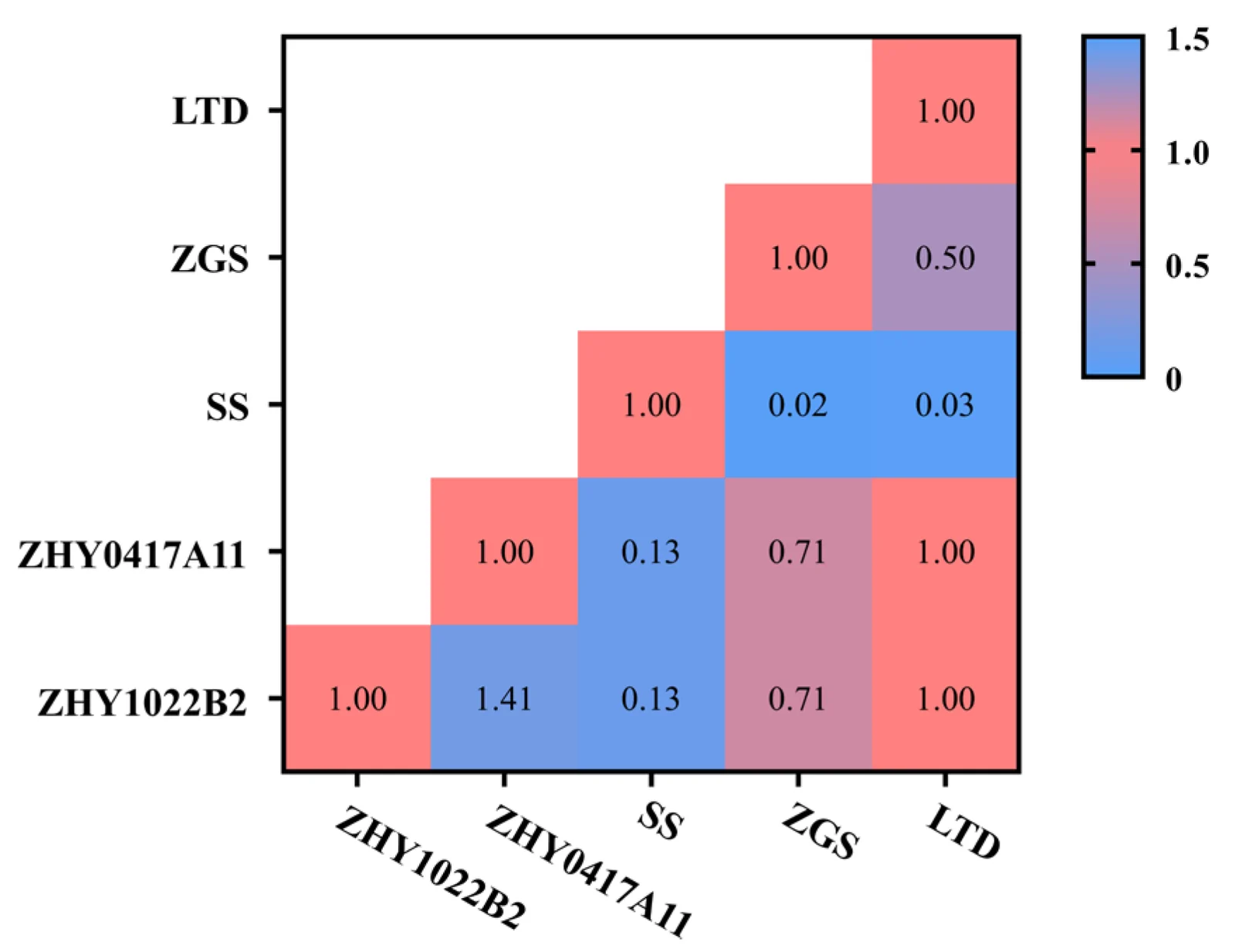
Antigenic correlation coefficients (R) between isolates ZHY1022B2/ZHY0417A11 and vaccine/epidemic strains, derived from cross-HI assays.

Cross-HI assays showed that ZHY1022B2 and ZHY0417A11 were antigenically closely related (HI titers of 9 and 12 log_2_, respectively; R = 1.0). In contrast, both isolates were antigenically divergent from the traditional vaccine strain SS, with an R value of 0.13 (well below the 0.5 threshold). Conversely, they remained closely related to the contemporary circulating strains ZGS and LTD, with R values of 0.71 and 1.00, respectively (Table 5 and Figure 5).

## 4. Discussion

H9N2 AIVs continue to threaten poultry production and public health, not only through direct economic losses but also via their capacity for reassortment and acquisition of mammalian-adaptive mutations [47,48,49]. In China, the B4.7.2 subclade of lineage B has become the dominant circulating H9N2 lineage in poultry [50], yet its evolutionary dynamics in quails remain underexplored.

Here we isolated and characterized two quail-origin H9N2 viruses that both fell into subclade B4.7.2 but were assigned to distinct subgroups—ZHY1022B2 in B4.7.2.2 and ZHY0417A11 in B4.7.2.1—with HA nucleotide and amino acid identities of 93.4% and 95.0% between them, and an average inter-subgroup genetic distance of 0.0642. Under the classification framework proposed by Fusaro et al. [21], these subgroups correspond to distinct fourth-level clades (B4.7.2.1 and B4.7.2.2). The recovery of these two viruses six months apart from different farms raises at least two possibilities: divergent evolution within the same geographic region, or independent introductions from external sources. Distinguishing between these scenarios will require expanded phylodynamic surveillance in quail populations. Regardless, these data suggest that H9N2 viruses circulating in quails are following evolutionary trajectories broadly similar to those in chickens, possibly reflecting ongoing intra-lineage divergence. The limited sample size (only two viruses from different farms and time points) precludes a definitive conclusion, and broader sampling is needed to clarify the underlying dynamics.

ZHY0417A11 displayed a complex reassortment pattern, with internal segments traceable to H3N3, H3N8, and H10N3 viruses (Figure 4). Of particular note is its M gene, which shares 99.32% nucleotide identity with a human-origin H3N8 strain (A/China/CSKFQ-22-5/2022)—providing molecular evidence of gene flow between avian and human-associated viruses. Given that H3N8 has been documented to cause human infections in China [51], the presence of such an M gene in a quail-origin isolate underscores the potential for quail-mediated reassortment to generate viruses with enhanced cross-species transmissibility. This observation aligns with recent reports from Xinjiang live-bird markets, where G57-genotype H9N2 viruses showed extensive lineage mixing and internal genes homologous to those of human-infecting strains [52], suggesting that inter-subtype gene exchange has become a recurring feature in contemporary H9N2 evolution. Moreover, quail populations are known to support the co-circulation and reassortment of multiple H9N2 lineages, and experimental studies have confirmed their capacity to generate reassortant progeny [15,53]. Together, these findings reinforce the view of quails as efficient mixing vessels that may facilitate the emergence of novel reassortants with elevated zoonotic risk.

The internal gene composition of both isolates—four internal genes plus HA and NA in the F/98-like lineage and M plus PB2 in the G1-like lineage—matches the G57 genotype that currently predominates in China. This genotype emerged in Hong Kong live-bird markets in the late 1990s and later became the dominant genotype in mainland China, serving as an internal gene donor for cross-species transmission events such as the H7N9 outbreak [2,5,54]. For ZHY1022B2, although its PB2 gene was acquired from an H6N8 virus, it clustered within the G1-like lineage; thus the overall genotype remains G57. Similarly, despite the complex origins of the ZHY0417A11 internal genes (H3N8, H3N3, and H10N3), their lineage assignments still conform to the G57 pattern (six F/98-like and two G1-like). The M genes of both isolates belong to the G1-like lineage; notably, the ZHY0417A11 M gene shows high homology to that of a human H3N8 virus, yet phylogenetic analysis places it within the same G1-like cluster. The G1-like M gene has been shown to enhance early infection and progeny virus production in chicken embryo fibroblasts and chickens, conferring stronger transcriptional and translational activity than the BJ/94-like M gene, and leading to more severe pathological damage and extrapulmonary spread [5]. The predominance of the G57 genotype likely reflects an optimal balance between replication efficiency, avian host adaptation, and cross-species infectivity [5]. The multisource reassortment observed here further suggests that the internal gene pool of the G57 genotype is undergoing ongoing exchange with emerging AIVs, potentially generating more adaptive and transmissible constellations that can be stably inherited by progeny viruses.

Molecular characterization revealed several features indicative of cross-species transmission potential. Within the HA RBD, both isolates carried H191N, A198V, and Q234L substitutions, which map to key helices and loops and have been experimentally shown to alter H9N2 host tropism [55]. Zhu et al. [55] demonstrated that A198V and Q234L enhance binding to human-type SAα2,6-Gal receptors and promote replication in human airway epithelial cells; Wan and Perez [6] further showed that Q234L alone shifts cellular tropism toward human airway epithelium, an effect potentiated by A198V [55]. The concurrent presence of these receptor-enhancing mutations in our isolates suggests a partial molecular basis for breaching the avian–mammalian receptor barrier. In addition, relative to the classical vaccine strains SS and F/98, both viruses had acquired substitutions at multiple HA antigenic sites (e.g., 90, 145, 153, 167, 168, 200, and 201), indicating ongoing antigenic drift that may challenge the efficacy of existing vaccines.

Some RBD substitutions differed between the two isolates: ZHY0417A11 carried N232H at the right edge, whereas ZHY1022B2 harboured A150T at the left edge (Table 2). These changes in side-chain polarity and charge distribution could modulate the local electrostatic microenvironment of the receptor-binding pocket, potentially influencing receptor recognition. However, this interpretation remains speculative and requires validation through molecular dynamics simulations or surface plasmon resonance assays. Both isolates retained N at position 163, rather than the I163T substitution previously linked to enhanced human-type receptor binding. Given the similar polarity and steric volume of N and T, the conserved N-X-S/T glycosylation motif at position 163 may partially compensate for the absence of I163T.

Both isolates carried a three-amino-acid deletion (Δ63–65) in the NA stalk, a feature previously associated with enhanced viral replication and pathogenicity in mammalian hosts [56,57]. This deletion is thought to increase NA access to sialic acid receptors, thereby facilitating viral release, and may act synergistically with HA receptor-binding changes to promote cross-species adaptation [48]. Its apparent fixation in these quail-origin viruses suggests a degree of pre-adaptation to mammalian hosts.

N-glycosylation site alterations can profoundly affect viral biology, antigenicity, and virulence. Both isolates possessed seven potential N-glycosylation sites but, relative to classical strains, lacked the site at position 218 and acquired a novel site at 313. Peng et al. [58] reported that gain of site 313 coupled with loss of site 218 may enhance binding to neutralizing antibodies, potentially impeding viral escape from homologous antiserum. Glycosylation changes may also stabilize HA trimers via steric hindrance, increasing infectivity. However, glycosylation-mediated modulation of antigenicity is a double-edged sword: newly acquired glycans can mask antigenic epitopes and reduce the neutralizing activity of vaccine-induced sera, potentially compromising vaccine efficacy. Differential NA glycosylation between the two isolates—ZHY0417A11 had a putative site at position 402 that was absent in ZHY1022B2—adds further complexity, particularly given that position 402 also falls within the hemadsorbing site [59].

Both isolates carried multiple substitutions in internal proteins previously linked to mammalian adaptation, enhanced virulence, or increased polymerase activity (Table 4). With respect to polymerase function, both retained the avian-signature E at PB2-627 rather than the mammalian-adaptive K, but harboured PA-K356R—a key mammalian marker that emerged in 2014 and has since persisted in avian H9N2 viruses in China [32]. Xu et al. showed that PA-356R can synergize with PB2-627K to promote mammalian infection; even in the absence of PB2-627K, the combination of PA-356R and PB2-588V, together with other adaptive substitutions such as PB2-89V and PB2-292V, is sufficient to enhance polymerase activity and viral yield in diverse cell lines [32,42]. PB1-3V and PB1-13P have also been implicated in host range expansion [36]. Thus, although neither isolate has acquired the canonical PB2-E627K marker—suggesting that cross-species transmission potential remains partially constrained—the presence of PA-356R and PB2-588V (the latter already widespread in both avian and human H9N2 isolates [52]) warrants continued monitoring. For immune evasion, both isolates shared NS1 substitutions 42S, 138F, and 149A, which attenuate type I interferon responses and enhance virulence in mammalian cells and mice [26,27,28,29]. The synergistic effects of these NS1 mutations, together with the polymerase-adaptive changes, highlight the multifactorial nature of mammalian adaptation. Regarding transmission and adaptive trade-offs, PB1-368V was detected exclusively in ZHY1022B2 and has been associated with enhanced airborne transmission in ferrets [38]. Conversely, residues such as PA-190S and PA-400P have been linked to reduced virulence in murine models [31], implying that these viruses may face adaptive trade-offs across divergent hosts. Overall, H9N2 mammalian adaptation is not driven by a single mutation but involves complex synergistic interplay among multiple proteins, including the polymerases (PA, PB1, PB2), NP, and NS1 [5,26]. The present data indicate a complex evolutionary landscape shaped by polymerase activity, immune modulation, transmission potential, and associated fitness costs. Elucidating the relative contributions of each substitution and their epistatic interactions will require further functional studies.

Serological analysis showed that both isolates were antigenically closely related to the contemporary circulating strains ZGS and LTD (R = 0.71–1.00), but divergent from the traditional vaccine strain SS (R < 0.5). This pattern is consistent with antigenic drift driven by sustained vaccine-induced immune pressure [50]. At equivalent antigen doses, ZHY0417A11 induced a four-fold higher antibody titer than ZHY1022B2, suggesting superior immunogenicity and a stronger humoral response. The presence of substitutions at multiple HA antigenic sites (e.g., 90, 145, 153, 167, 168, 200, and 201) relative to SS and F/98 provides a molecular basis for this drift. Such antigenic divergence may compromise the efficacy of existing inactivated vaccines, weaken herd immunity, and thereby facilitate sustained viral circulation and cross-regional transmission [47].

The persistent circulation of H9N2 in poultry and the inter-lineage reassortment of its internal gene pool reflect an ongoing evolutionary process that may favour enhanced mammalian adaptation [60]. Quail farming practices often bring together viruses of multiple avian origins, creating conditions for gene exchange. Should a novel reassortant with cross-species transmission potential emerge in quail populations, it could spread rapidly through live-bird supply chains and pose a risk at the animal–human interface [60]. These considerations support the systematic inclusion of quail farms in national and regional AIV active surveillance networks, with enhanced tracking of viral genetic diversity and reassortment events in this host. We recommend integrating quail farms into provincial surveillance frameworks, annually assessing antigenic variation within subclade B4.7.2, and prioritising screening for complex reassortment patterns and mammalian-adaptive mutations in live-bird trade chains.

Several limitations should be noted. First, the study is based on only two isolates, limiting the generalisability of our conclusions. Second, the absence of in vivo pathogenicity studies and the restricted panel of reference antisera constrain our inferences regarding virulence and antigenic breadth. Third, although the two isolates were recovered from different farms six months apart, we cannot definitively distinguish between local divergence and independent introductions without broader phylodynamic surveillance. With these caveats, our findings should be interpreted as an initial molecular snapshot of quail-origin H9N2 viruses rather than a comprehensive evolutionary portrait.

## 5. Conclusions

This study provides molecular evidence of subclade B4.7.2 H9N2 viruses in Chinese quails, identifying two distinct subgroups (B4.7.2.1 and B4.7.2.2). Strain ZHY0417A11 exhibited complex reassortment, with internal genes derived from H3N8, H3N3, and H10N3 viruses, including an M gene closely related to a human-origin H3N8 strain. Both isolates carried mammalian-adaptive mutations and were antigenically divergent from the vaccine strain SS. These findings support the role of quails as viral mixing vessels and argue for enhanced surveillance and candidate vaccine development. Given the limited sample size, our conclusions remain preliminary and require validation through expanded epidemiological and functional studies. More systematic monitoring of quail populations is needed to clarify their contribution to AIV ecology and to assess potential zoonotic risks.

## Figures and Tables

**Table 1:** Influenza viruses with the highest nucleotide identity to ZHY1022B2 and ZHY0417A11.

Virus	Gene	Closest Strain	Identity	Accession No.
ZHY1022B2	PB2	A/chicken/China/Guangdong_01/2022 (H6N8)	98.46%	ON627703
	PB1	A/chicken/Anhui/12.23BBZGS14-C/2021 (H9N2)	98.77%	PV372069
	PA	A/chicken/Anhui/12.23BBZGS14-C/2021 (H9N2)	98.70%	PV372841
	HA	A/chicken/Hunan/3.24 YYWLP-K2/2023 (H9N2)	98.93%	PV374130
	NP	A/chicken/Hunan/3.24 YYWLP-K2/2023 (H9N2)	99.67%	PV375167
	NA	A/chicken/Hunan/3.24 YYWLP-K2/2023 (H9N2)	98.69%	PV375856
	M	A/chicken/Yunnan/4-15XGLLXY0018-O/2022 (H9N2)	99.29%	PV381459
	NS	A/chicken/Hunan/3.24 YYWLP-K2/2023 (H9N2)	99.44%	PV377864
ZHY0417A11	PB2	A/chicken/China/YC01/2023 (H3N3)	98.73%	PP998321
	PB1	A/chicken/Guangzhou/4463/2021 (H3N8)	97.93%	OQ291939
	PA	A/chicken/Henan Shangqiu/SQ2049/2023 (H3N3)	98.37%	PP758471
	HA	A/environment/Xiamen/01/2021 (H9N2)	97.68%	ON856628
	NP	A/chicken/Anhui/11.30ZGS1-C/2021 (H9N2)	98.03%	PV378649
	NA	A/chicken/Hunan/07.02YYWLP28-O/2022 (H9N2)	98.27%	PV375521
	M	A/China/CSKFQ-22-5/2022 (H3N8)	99.32%	ON627821
	NS	A/chicken/Jiangsu/JS10/2022 (H10N3)	97.37%	PQ661124

**Table 2:** Key amino acid residues in the HA receptor-binding domain (H9 numbering).

Virus	HA Receptor-Binding Domain								
	Left Edge (146–150)	109	161	163	191	198	202	203	Right Edge (232–237)
ZHY1022B2	GTSNT	Y	W	N	N	V	L	Y	NGLMGR
ZHY0417A11	GTSNA	Y	W	N	N	V	L	Y	HGLMGR
BJ/94	GTSKA	Y	W	T	N	V	L	Y	NGQQGR
SS	GTSKA	Y	W	T	N	A	L	Y	NGQQGR
F/98	GTSKA	Y	W	T	N	A	L	Y	NGQQGR
Y280	GTSKA	Y	W	T	N	T	L	Y	NGLQGR
G1	GISRA	Y	W	T	H	E	L	Y	NDLQGR

**Table 3:** Key amino acid residues in the HA antigenic regions (H9 numbering).

Virus	HA Antigenic Sites						
	90	145	153	167	168	200	201
ZHY1022B2	E	D	G	G	N	R	T
ZHY0417A11	D	D	G	N	N	R	T
BJ/94	G	S	D	N	A	T	N
SS	G	S	D	N	A	T	N
F/98	G	S	D	N	A	T	N
Y280	G	S	D	N	A	T	N
G1	E	T	D	G	F	T	N

**Table 4:** Key amino acid residues in the internal proteins of the influenza viruses and their associated functions.

Protein	Position	Associated Function	Reference
NS1	42S	Increased virulence in mice and reduced antiviral response	[26]
	138F	Increased replication in mammalian cells and reduced interferon response	[27]
	149A	Increased virulence in chickens and reduced interferon response	[26,28]
NP	105V	Increased virulence in chickens	[29]
	184K	Increases replication in avian cells and virulence in chickens, increases the IFN response	[30]
PA	190S and 400P	Reduced virulence in mice	[31]
	356R	Enhanced mammalian replication and pathogenicity	[32]
	224P and 383D	Increased polymerase activity in mammalian and avian cell lines	[33,34]
	672L	Affects viral aerosol transmission properties	[35]
PB1	3V	Increased polymerase activity and viral replication in avian and mammalian cell lines	[36]
	13P	Enhanced mammalian adaptation	[37]
	368V	Enhanced transmission in ferrets	[38]
PB2	89V	Increased mammalian pathogenicity and virulence	[39,40]
	292V	Increased polymerase activity in mammalian cell lines and increased virulence in mice	[41]
	588V	Increased polymerase activity and replication in mammalian and avian cell lines and increased virulence in mice	[42]
	627E	Increased virulence in chickens	[43,44,45,46]

**Table 5:** Cross-HI antibodies against H9N2 influenza viruses in HI assays.

Antigen	Antibody Titers (log_2_) of Indicated Antisera				
	ZHY1022B2	ZHY0417A11	SS	ZGS	LTD
ZHY1022B2	9	12	7	11	10
ZHY0417A11	9	11	9	11	11
SS	7	7	11	5	4
ZGS	9	11	7	12	10
LTD	9	10	7	10	10

## Data Availability

The genome sequences of strain ZHY1022B2 have been submitted to GenBank under accession numbers PZ146727, PZ270046, PZ270048, PZ270044, PZ270042, PZ270050, PZ270052, and PZ270054. Those of strain ZHY0417A11 are available under accession numbers PZ146728, PZ270047, PZ270049, PZ270045, PZ270043, PZ270051, PZ270053, and PZ270055.

## Supplementary Materials

The following supporting information can be downloaded at: https://www.mdpi.com/article/10.3390/v18080919/s1, Table S1: Influenza viruses with the highest amino acid identity to ZHY1022B2 and ZHY0417A11.

## Abbreviations

The following abbreviations are used in this manuscript:

AIV	Avian influenza virus
HA	Hemagglutinin
NA	Neuraminidase
PB2	Polymerase basic 2
PB1	Polymerase basic 1
PA	Polymerase acidic
NP	Nucleoprotein
M	Matrix
NS	Non-structural
HI	Hemagglutination inhibition
RBD	Receptor-binding domain
SPF	Specific pathogen-free
RT-PCR	Reverse transcription polymerase chain reaction
RT-qPCR	Real-time quantitative reverse transcription polymerase chain reaction
WOAH	World Organisation for Animal Health
