# Supplementary material for: Genetic Diversity, Reassortment Patterns, and Antigenic Characterization of Two H9N2 Avian Influenza Viruses Isolated from Quails in China
Source: Viruses. 2026 Aug 21;18(8):919. doi: 10.3390/v18080919 (PMC13517541; doi:10.3390/v18080919)
Supplement: Supplementary file 1 [file viruses-18-00919-s001.zip › viruses-4476868-supplementary.pdf]

Supplementary Table

Table S1. Influenza viruses with the highest amino acid identity to ZHY1022B2 and ZHY0417A11.

Virus	Protein	Closest strain	Identity	Accession no.
ZHY1022B2	PB2	A/chicken/Chongqing/4.3CQSKS1-C/2022 (H9N2)	98.95%	XQM59459
	PB1	A/chicken/China/4-1JN/2022 (H9N2)	99.74%	WMV93708
	PA	A/chicken/Anhui/10.29BBZGS25-O/2021 (H9N2)	98.60%	XQM58131
	HA	A/chicken/Hunan/3.24 YYWLP-K2/2023 (H9N2)	99.82%	XQM51912
	NP	A/chicken/China/2-3-1/2022 (H9N2)	99.60%	WMV93819
	NA	A/chicken/Hunan/02.19 YYWLP-O11/2022 (H9N2)	98.50%	XQM53558
	M	A/chicken/China/811/2016 (H9N2)	99.60%	QAU22069
	NS	A/chicken/Fujian/SD070/2017 (H9N2)	96.62%	ATO98089
ZHY0417A11	PB2	A/chicken/China/16/2023 (H3N3)	98.95%	WMM65683
	PB1	A/chicken/Huizhou/168/2022 (H3N8)	98.41%	WBW07563
	PA	A/chicken/China/HN0120/2023 (H3N3)	99.02%	XBD72880
	HA	A/chicken/China/YL/2022 (H9N2)	97.68%	WRK30336
	NP	A/chicken/China/KM216/2022 (H9N2)	100.00%	WRK34329
	NA	A/chicken/Hunan/07.02YYWLP28-O/2022 (H9N2)	99.35%	XQM53303
	M	A/environment/Shanghai/TY01/2013 (H7N9)	99.21%	AHF20562
	NS	A/chicken/Fujian/SD070/2017 (H9N2)	97.05%	ATO98089
